# Aptamer-mediated survivin RNAi enables 5-fluorouracil to eliminate colorectal cancer stem cells

**DOI:** 10.1038/s41598-017-05859-z

**Published:** 2017-07-19

**Authors:** Hadi AlShamaileh, Tao Wang, Dongxi Xiang, Wang Yin, Phuong Ha-Lien Tran, Roberto A. Barrero, Pei-Zhuo Zhang, Yong Li, Lingxue Kong, Ke Liu, Shu-Feng Zhou, Yingchun Hou, Sarah Shigdar, Wei Duan

**Affiliations:** 1 School of Medicine and Centre for Molecular and Medical Research, Deakin University, 75 Pigdons Road, Waurn Ponds, Victoria, 3216 Australia; 2 School of Nursing, Zhengzhou University, Zhengzhou, Henan Province 450001 China; 3 Centre for Comparative Genomics, Murdoch University, 90 South Street, Murdoch, WA 6150 Australia; 4 Suzhou GenePharma, 199 Dongping Street, Suzhou, 215123 China; 5 Cancer Care Centre, St George Hospital and St George and Suthland Clinical School, University of New South Wales (UNSW), High Street, Kensington, NSW 2052 Australia; 6 Deakin University, Institute for Frontier Materials, 75 Pigdons Road, Waurn Ponds, Victoria, 3216 Australia; 7 College of Life Sciences, Sichuan University, No.24 South Section 1, Yihuan Road, Chengdu, 610041 P. R. China; 8 Department of Bioengineering and Biotechnology, College of Chemical Engineering, Huaqiao University, 668 Jimei Avenue, Xiamen, Fujian 361021 China; 9 Center for Qinba Region’s Sustainable Development, Shaanxi Normal University, No.199, South Chang’an Road, Xi’an, Shaanxi 710062 China

## Abstract

The development of chemoresistance and inability in elimination of cancer stem cells are among the key limitations of cancer chemotherapy. Novel molecular therapeutic strategies able to overcome such limitations are urgently needed for future effective management of cancer. In this report, we show that EpCAM-aptamer-guided survivin RNAi effectively downregulated survivin both in colorectal cancer cells *in vitro* and in a mouse xenograft model for colorectal cancer. When combined with the conventional chemotherapeutic agents, the aptamer-guided survivin RNAi was able to enhance the sensitivity towards 5-FU or oxaliplatin in colorectal cancer stem cells, increase apoptosis, inhibit tumour growth and improve the overall survival of mice bearing xenograft colorectal cancer. Our results indicate that survivin is one of the key players responsible for the innate chemoresistance of colorectal cancer stem cells. Thus, aptamer-mediated targeting of survivin in cancer stem cells in combination with chemotherapeutic drugs constitutes a new avenue to improve treatment outcome in oncologic clinics.

## Introduction

Cancer stem cells are regarded as the ‘roots of cancer’ and therefore are an attractive therapeutic target to inhibit tumour growth, expansion and metastasis^1, 2^. Current approaches in treating cancer patients include surgical resection with chemotherapy or radiotherapy. Although they are effective in eliminating the majority of the cancer cells, their inability to eliminate the cancer stem cell population remains a limitation for current regimen for cancer treatment^3–7^. Indeed, cancer stem cells have been shown to be resistant to conventional anticancer drugs through a variety of mechanisms including overexpression of ABC transporters, active DNA-repair capacity, overexpression of antiapoptotic proteins, and elevated autophagy^8–10^.

5-fluorouracil (5-FU) is a chemotherapeutic drug widely used for the treatment of colorectal cancer and is often included in the first line treatment. However, cancer stem cells in colorectal cancer have been shown to be resistant to 5-FU as well as other chemotherapeutic drugs such as oxaliplatin and irinotecan^11^. Furthermore, the use of these drugs could lead to the enrichment of cancer stem cells with high tumourigenic capacities in a number of solid cancers^11–13^. Thus, despite the efficacy of these drugs against bulk cancer cells, they remain mostly ineffective against cancer stem cells^14, 15^. We hypothesized that aptamer-mediated survivin RNAi enables 5-fluorouracil to eliminate colorectal cancer stem cells and improves the survival of the mice bearing xenograft tumour.

Aptamers are single stranded DNA or RNA that fold into defined 3-D structure and bind to their targets specifically. As ‘chemical antibodies’, aptamers offer significant advantages over antibodies in terms of smaller size, lower immunogenicity, increased stability, ease of synthesis and modification^16–19^. As a targeting module, aptamers allow for improved delivery of drugs to tumour cells while minimizing the side effects often associated with the drugs. Our laboratory has developed the first RNA aptamers that target the cancer stem cell markers EpCAM. Upon binding to target cells, our EpCAM aptamers undergo receptor-mediated endocytosis which allows for controlled release of drugs intracellularly^20, 21^. Here we describe the use of the EpCAM aptamer in delivering siRNA to target survivin expression in order to eliminate colorectal cancer stem cells.

Survivin, a member of inhibitors of apoptosis proteins family, is an attractive target for anticancer therapies for its role in inhibiting apoptosis and its close association with stem cells^22, 23^. In addition to its critical role in inhibiting cell death, survivin is expressed at low levels or absent in terminally differentiated healthy cells, but its expression is significantly elevated in human tumours and its overexpression is further enhanced when exposed to traditional anticancer treatments^24–26^. However, the role of survivin in the development of chemoresistence in solid tumours remains largely unexplored. Here we demonstrate that by silencing survivin expression in colorectal cancer stem cells using an aptamer-siRNA chimera, the innate chemoresistance to traditional chemotherapeutic agents in colorectal cancer stem cells was reversed, transforming an old anticancer drug into a cancer stem cell killer.

## Results

### Effective downregulation of survivin via EpCAM Aptamer-guided RNAi in colorectal cancer cells

**Figure 1:**
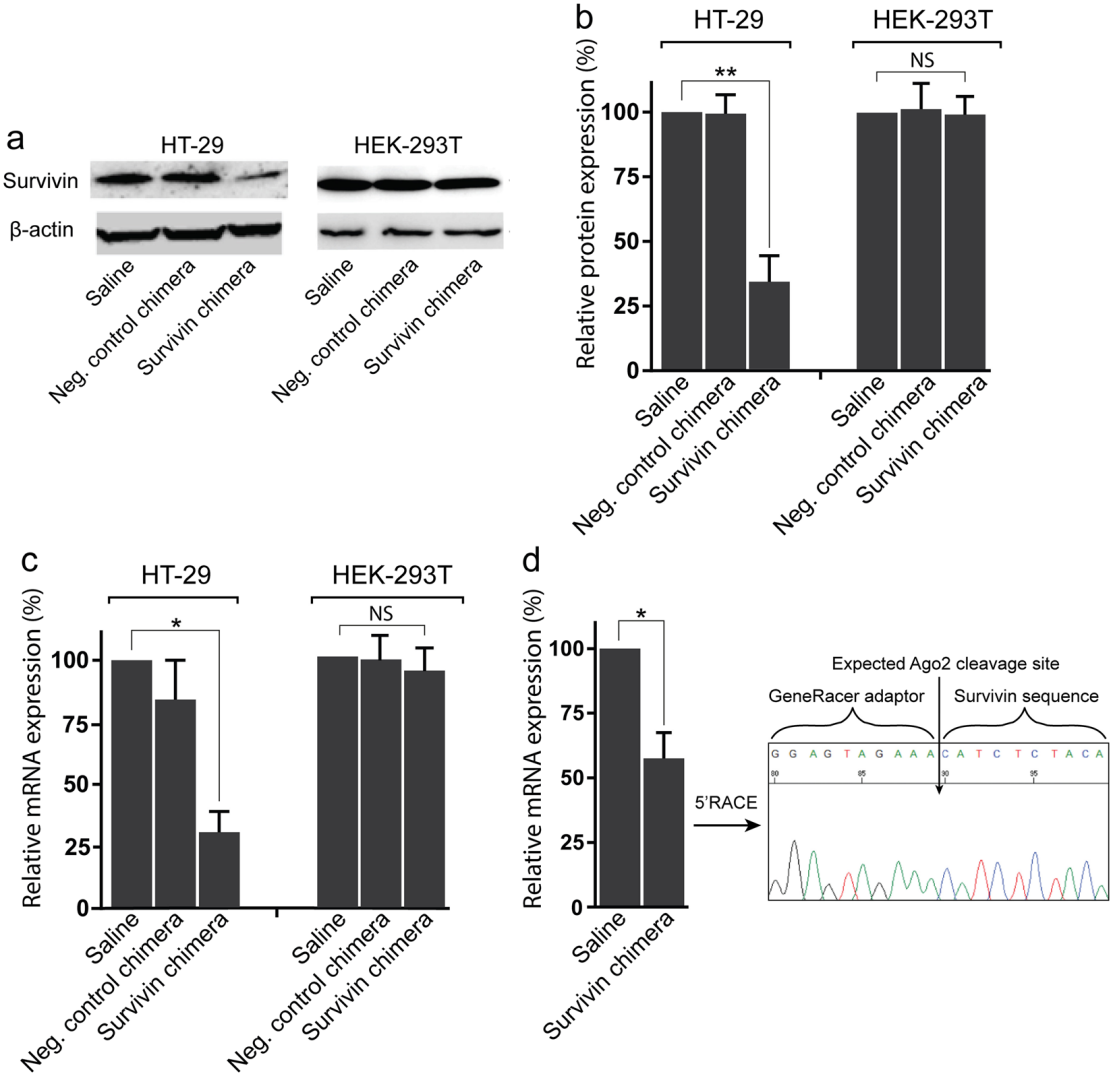
EpCAM aptamer-guided RNAi effectively silenced survivin. (**a**) Specificity and efficacy of EpCAM-aptamer guided RNAi in knocking down survivin mRNA. Chimera or negative control chimera were incubated with HT-29 or HEK-2913T cells for 24 hours and the total RNA was extracted for qRT-PCR analysis of survivin mRNA levels. GAPDH was used as an internal control. (**b**,**c**) HT-29 Tumour-bearing mice were treated with 2 nmol/mouse of PEG-labelled chimera for 48 hours. The tumours were collected for RNA extraction followed by qRT-PCR analysis of survivin mRNA expression (**b**) and 5′RACE assay (**c**). (**d**) Effective downregulation of survivin protein via EpCAM aptamer-guided RNAi. Chimera or negative control chimera were incubated with HT-29 or HEK-2913T cells for 48 hours and the survivin protein levels were analyzed using Western blot analysis. β-actin was used as a loading control. (**e**) The bar graph shows the survivin protein levels in various treatment groups. Data shown are means ± SEM, n = 3. *p < 0.05, **p < 0.005. NS, no statistically significant difference.

In this study, we targeted survivin expression in colorectal cancer stem cells using a chimeric structure composed of an EpCAM-specific RNA aptamer as the binding moiety and a survivin siRNA sequence (Supp. Fig. 1). The efficacy of EpCAM aptamer-survivin siRNA chimera in the downregulation of survivin was initially tested on EpCAM-positive colorectal cancer cell line HT-29 with the EpCAM-negative cell line HEK-293T and a survivin RNAi-deficient variant chimera as negative controls (Supp. Fig. 1)^27^. Both cell lines were treated with 20 nM of the chimera or the negative control chimera for 24 hours or 48 hours in the absence of any transfection agent and subjected to qRT-PCR and Western analysis, respectively. As shown in Fig. 1a,d,e, the treatment of chimera resulted to a ~70% knockdown of both survivin mRNA and protein levels in EpCAM-positive HT29 cells but not in the EpCAM-negative control HEK293T cells. Having demonstrated the efficacy and selectivity of EpCAM-directed survivin RNAi *in vitro*, we proceeded to evaluate the chimera-mediated survivin RNAi *in vivo*. To this end, a 20-kDa polyethylene glycol (PEG) was attached to the chimera to increase circulatory half-life of the chimera in mice (Supp. Fig. 1)^27^.

Next, we studied efficacy and mode of action of aptamer-guided survivin RNAi *in vivo*. The HT29 xenograft tumour-bearing NOD/SCID mice were treated with a single dose of 2 nmol/mouse of the PEG-chimera *i.v*. Forty-eight hours after the treatment, the tumours were removed and the survivin mRNA content in the treated HT29 xenograft tumour was evaluated. As shown in Fig. 1b, a single dose of 2 nmol/mouse of the chimera resulted in the reduction of survivin mRNA in the xenograft tumour by ~40%. Furthermore, a 5′RACE assay on the RNA extracted from the treated xenograft tumour suggests specific survivin mRNA cleavage at the expected siRNA cut site through the Argonaute2 (Ago2) mechanism (Fig. 1c). Taken together, these data indicate that aptamer-siRNA chimera is able to effectively downregulate survivin in EpCAM-positive colorectal cancer cells both *in vitro* and *in vivo* in the absence of transfection reagent.

### Survivin silencing combined with 5-FU target colorectal cancer stem cells *in vitro*

Next, we explored if the downregulation of survivin would lead to the increased sensitivity of colorectal cancer stem cells to standard chemotherapy agents, such as 5-FU, *in vitro* and *in vivo*. For the evaluation of the elimination of colorectal cancer stem cells rather than the bulk cancer cells, we used self-renewal and tumour-formation as the key experimental endpoints as such properties reflect the defining functional characteristics of the cancer stem cell population^28–30^.

**Figure 2:**
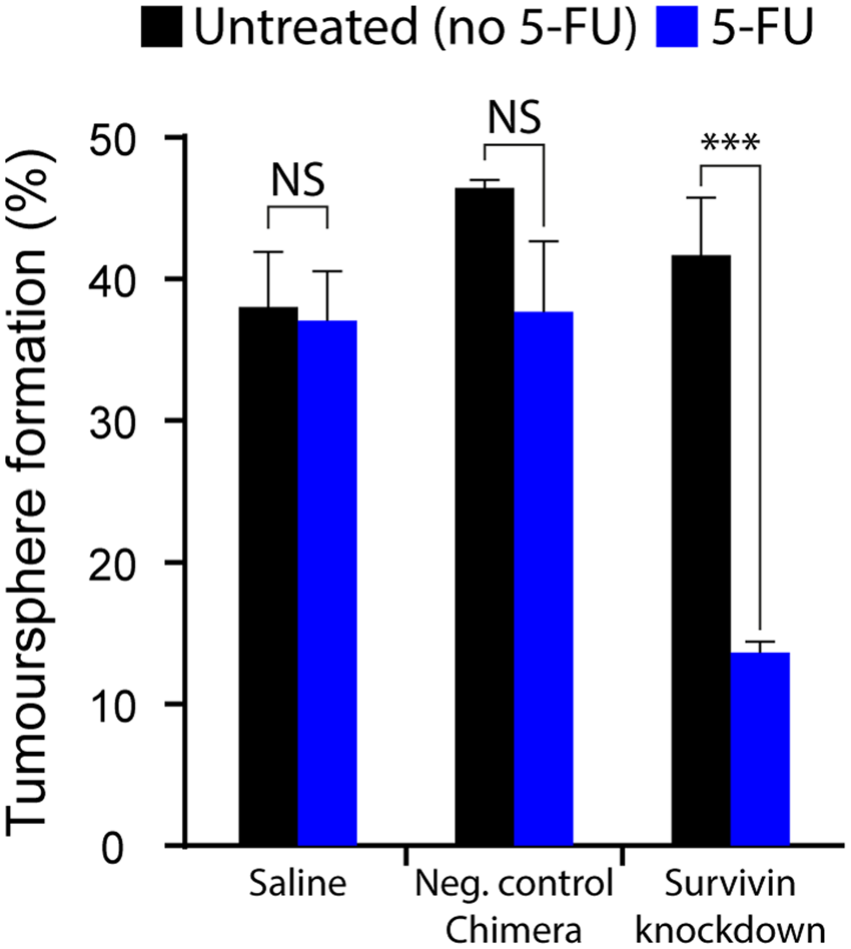
EpCAM aptamer mediated survivin knockdown transforms 5-FU into a colorectal cancer stem cell killer. Colorectal cancer HT-29 cells were first treated with EpCAM aptamer-survivin siRNA chimera for 24 hours followed by treatment with 2 μM 5-FU for 5 days. Tumoursphere formation assay was used to examine the self-renewal capacities of treated cells. The bar graph shows the estimated stem cell frequencies. Data shown are means ± SEM, n = 3. ***p < 0.001. NS, no statistically significant difference.

For an *in vitro* assessment of the self-renewal capacity of cancer stem cells, HT-29 cells were treated with the EpCAM aptamer-survivin siRNA chimera or negative control chimera, followed by further incubation in the presence or absence of 2 µM 5-FU for 5 days. A tumoursphere formation assay was used to enumerate the number of tumourspheres formed. As shown in Fig. 2 and Suppl. Fig. 2, a statistically significant 3-fold decrease in self-renewal was observed when HT-29 cells were treated with combined survivin chimera and 5-FU. In contrast, the treatment with either chimera or 5-FU alone, negative control chimera or saline did not lead to significant decrease in self-renewal capacity of the HT-29 cells *in vitro*. These data indicate that only the combinatorial treatment of survivin knockdown and 5-FU but not the mono-treatment regimen or the negative controls, effectively impaired the self-renewal capacity of HT-29 *in vitro*. To further verify if the combinatorial treatment leads to the impairment of tumourigenicity of colorectal cancer stem cells *in vivo*, we performed tumour xenograft experiments under the limiting dilution conditions. Treated HT-29 cells were implanted subcutaneously into the immunodeficient NOD/SCID mice at limiting dilutions with an inoculation cell dose between 1 × 10^3^ to 5 × 10^4^ cells per mouse. As shown in Table S1, there was no significant decrease in tumourigenicity potential in HT-29 cells treated with 5-FU alone or with the negative control chimera. Similarly, targeting survivin expression by mono-treatment with the chimera had little impact on the tumour-forming potential compared to that of saline-treated control (Table S1). In contrast, a significant decrease in HT-29 tumour formation for HT-29 cells treated with the combinatorial regimen was evident, indicating that although survivin knockdown as a mono-treatment is not sufficient to eliminate HT-29 cancer stem cells, the combination with survivin RNAi with classical chemotherapeutic drugs, such as 5-FU, is able to eliminate colorectal cancer stem cells. The downregulation of survivin in empowering traditional chemotherapeutic agents to eliminate colorectal cancer stem cell is not limited to 5-FU as the combined treatment with aptamer-survivin siRNA chimera and oxaliplatin had a similar efficacy in the impairment of self-reviewal capacity of HT-29 colorectal cancer stem cells (Supp. Fig. 3).

### Combinatorial survivin knockdown and 5-FU treatment *in vivo* eliminates colorectal cancer stem cells

To evaluate if the combinatorial treatment of chimera and 5-FU leads to the elimination of HT-29 cancer stem cells *in vivo*, we treated HT-29 xenograft tumour-bearing NOD/SCID mice using PEG-aptamer-siRNA chimera with or without 5-FU. Mice bearing 60 mm^3^ HT-29 tumours received *i.v*. injections of aptamer-siRNA chimera (2 nmol/mouse) on days 1, 3, and 5 with or without 30 mg/kg of 5-FU injections on days 3, 5, and 7. Forty-eight hours after the last treatment, tumours were removed (Supp. Fig. 4) and single-cell suspensions were subjected to tumoursphere formation assay to analyze their self-renewal capacities. As shown in Table 1 and Supp. Fig. 5, xenograft tumours from mice that received chimera in the absence of 5-FU showed no significant change in self-renewal when compared with saline-treated mice. Similarly, 5-FU alone or control chimera had no significant impact on self-renewal either. Interestingly, the HT29 xenograft tumours from the mice that received aptamer-survivin siRNA chimera in combination with 5-FU showed significant reduction in self-renewal (Table 1).

**Table 1:** Elimination of cancer stem cells via combined *in vivo* survivin knockdown and 5-FU treatment *in vivo*.

Treatment group	Number of cells/well					Estimated stem cell frequency	95% confidence interval
	50	20	10	5	1		
Saline	4/10	3/10	1/10	1/10	0/10	1.3%	0.65–2.5%
Saline + 5-FU	5/10	1/10	1/10	0/10	0/10	0.99%	0.47–2.1%
Neg. chimera control + 5-FU	3/9	1/7	1/10	0/10	0/10	0.76%	0.32–1.8%
Chimera	3/7	1/10	1/10	0/10	0/10	0.81%	0.30–1.9%
Chimera + 5-FU	1/10	1/10	0/10	0/10	0/10	0.24%	0.06–0.97%

**Figure 3:**
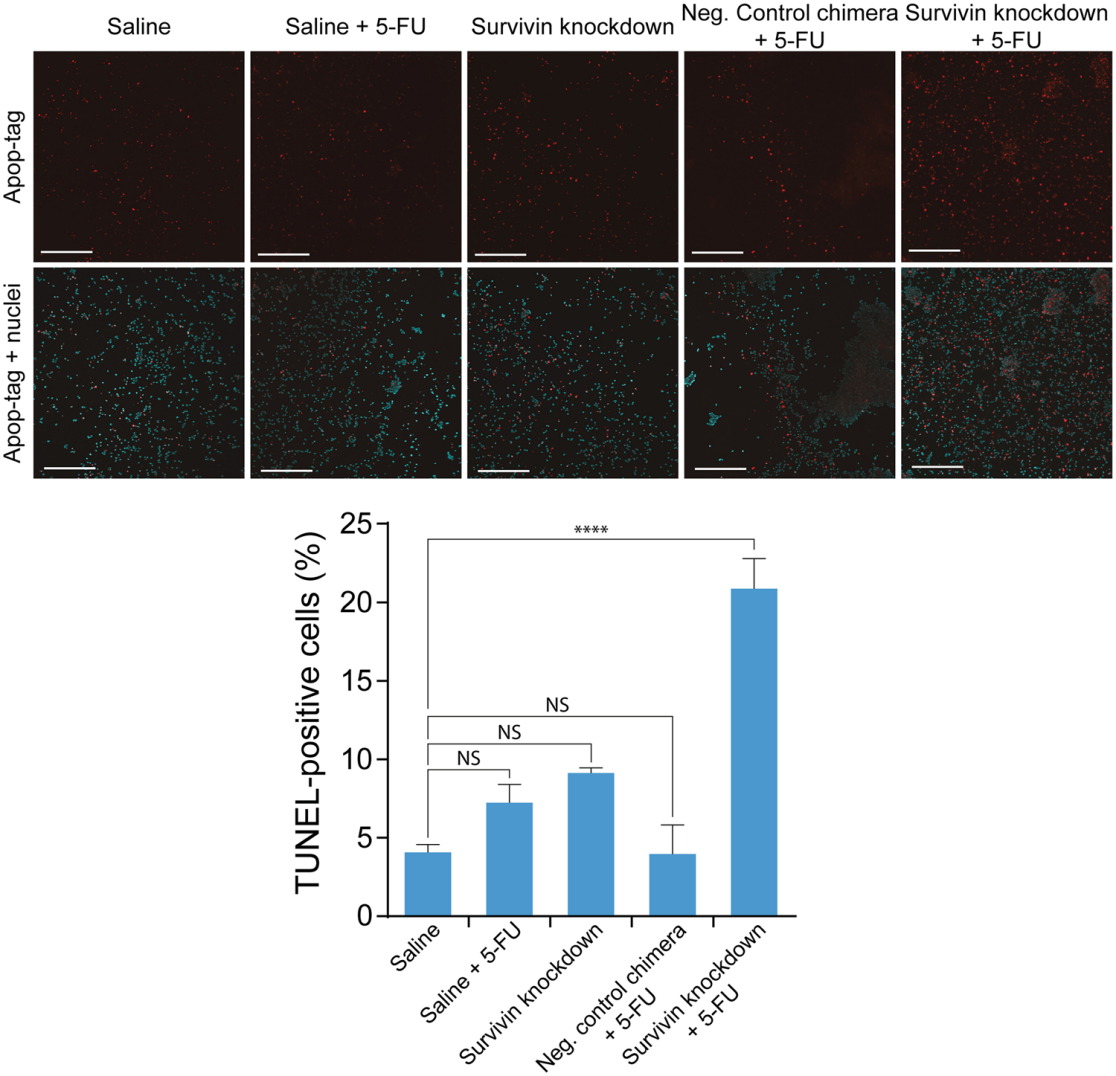
Survivin knockdown *in vivo* enhances 5-FU-induced apoptosis in HT-29 tumour cells. (**a**) Representative images of TUNEL apoptosis assay on dissociated HT-29 xenograft tumours after *in vivo* treatment with chimera and 5-FU. NOD/SCID mice bearing HT-29 tumours (60 mm^3^) were treated intravenously with 3 injections of 2 nmol/mouse of chimera with or without 3 additional treatment of 30 mg/kg of 5-FU. Two days after the final treatment, tumours were dissociated by collagenase digestion and subjected to TUNEL apoptosis assay. (**b**) Percentage of apoptotic cells in treated tumours. Data shown are means ± SEM, n = 3. ****p < 0.001. NS, no statistically significant difference.

As 5-FU is known to kill cancer cells via the induction of apoptosis, we proceeded to investigate the mechanism of action in colorectal cancer stem cells upon combinatorial treatment of chimera and 5-FU *in vivo*. For this purpose, dissociated tumour cells and tumour sections from mice were fixed and underwent TUNEL staining to enumerate apoptotic cells (Fig. 3 and Supp. Fig. 6). Tumours from mice that received mono-treatments of chimera or 5-FU were found to have 7.23% ± 2.038 and 9.132% ± 0.5787 apoptotic cells, respectively (Fig. 3a,b). Similarly, tumours from mice that received negative control chimera with 5-FU treatment showed 3.975% ± 3.213 of the population to have undergone apoptosis. In contrast, tumours from mice that received combinatorial treatment of chimera and 5-FU displayed enhanced apoptosis as 20.88% ± 3.32 of the tumour cell population had undergone apoptosis. Thus, EpCAM aptamer-guided RNAi of survivin enhanced the 5-FU-mediated cell death in colorectal cancer cells via enhanced apoptosis. Furthermore, tumour sections stained for Ki-67 demonstrated 4-fold decrease of tumour cell proliferation when treated with chimera and 5-FU combinatorial treatment (Supp. Fig. 7).

### EpCAM-aptamer guided *in vivo* survivin RNAi enhanced treatment efficacy of 5-FU

**Figure 4:**
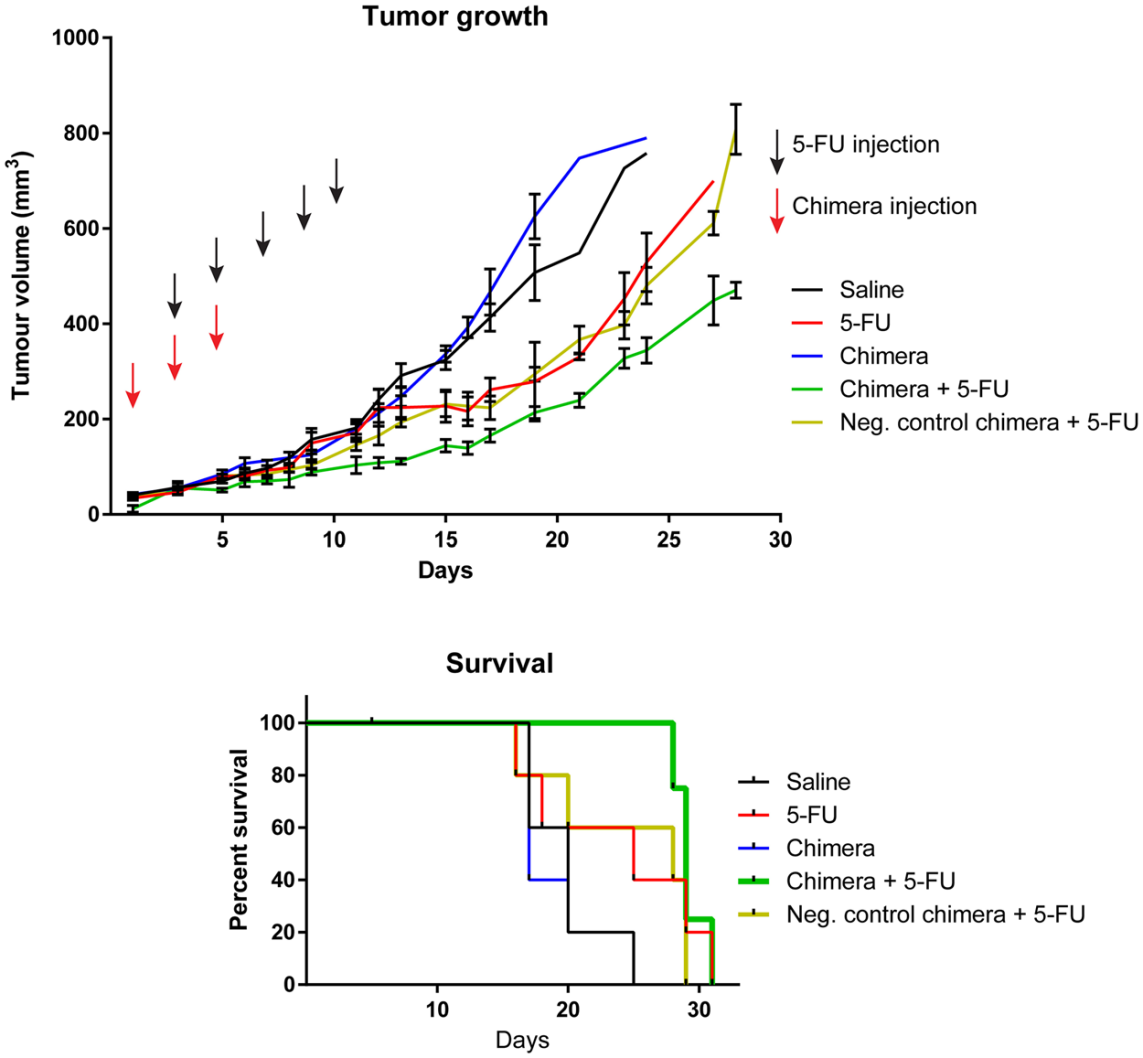
Combined chimera and 5-FU treatments improves therapeutic outcome in HT-29 tumour-bearing mice. (**a**) The graph represents the tumour volume in mice in response to various treatments as indicated. (**b**) Survival rate of mice observed over the course of various treatments. Data shown are mean ± SEM, n = 4–5.

Next, we examined if the suppression of self-renewal and increased apoptosis conferred by the combinatorial treatment of aptamer-guided survivin siRNA and 5-FU *in vivo* would result in an improved treatment efficacy in mice bearing HT-29 xenograft tumour. NOD/SCID mice bearing HT-29 xenograft colorectal tumours were treated with 2 nmol/mouse of PEG-chimera or negative control chimera on days 1, 3, and 5, with or without 30 mg/kg 5-FU on days-3, 5, 7, 9, and 11. The changes in tumour volume were monitored daily until the animals reached their experimental endpoints. The mice that underwent mono-treatment with the chimera showed no signs of reduced rate of tumour growth (Fig. 4a) nor improvement in survival (Fig. 4b) when compared to the saline control group. The tumour-bearing mice that received 5-FU mono-treatment with or without the negative control chimera showed slower tumour growth compared to the saline group (Fig. 4a). In contrast, the combinatorial treatment of chimera and 5-FU led to the significantly slower rate of tumour growth compared to the mice that received 5-FU treatment with or without negative control chimera (Fig. 4a), and improved overall survival within the first month of treatment (Fig. 4b).

## Discussion

Survivin is known to be overexpressed after chemotherapy and radiotherapy in many solid cancers including colorectal cancer, and such overexpression is largely associated with drug resistance, tumour aggressiveness and poor prognosis^31^. Our experience with chemotherapeutic drugs in the past 60 years indicates that despite the limited success in eliminating the bulk cancer cells, these anti-cancer agents, such as 5-FU, remain largely ineffective against cancer stem cells^14, 15^. Here we describe the use of an oligonucleotide chimera made up of an EpCAM-specific aptamer and a survivin siRNA to downregulate survivin in both the bulk and colorectal cancer stem cells without the aid of transfection agent or other delivery vehicles such as viral vectors or nanodrug carriers. When combined with the co-treatment with conventional chemotherapy drugs, the aptamer-guided survivin RNAi resulted in enhanced sensitivity of the cancer stem cells towards 5-FU or oxaliplatin. The aptamer-siRNA chimera used here was previously established to specifically target EpCAM-expressing cancer cells, undergo receptor-mediated endocytosis upon binding to target cells, and engage cell’s endogenous RNAi machinery^27^. Notably, EpCAM is overexpressed on both bulk cancer cells and cancer stem cells^32, 33^. We purposely engineered an EpCAM aptamer with a moderate dissociation constant so that it will preferentially be enriched at the site of tumour as cancer cells express up to 1000-fold higher EpCAM than normal epithelial cells^34, 35^. Therefore, the EpCAM aptamer-survivin siRNA chimera used in this study can effectively target both the cancer stem cells and non-cancer stem cells *in vivo*. Such dual targeting capacity is critical for the success of clinical oncology since it has been established that cancer stem cells and non-cancer stem cell may interconvert, and survivin has been shown to be involved in the regulation of such interconversion^36, 37^. It is thus imperative not to limit the targeted therapies against cancer stem cells but also target the non-cancer stem cell population simultaneously. To our knowledge, this fully synthetic aptamer-siRNA chimera is the shortest of its kind ever developed, providing relative ease for large scale chemical synthesis and modification^27^.

Depending on the experimental systems and approaches used, inhibition of survivin function alone could lead to decreased tumour cell viability, increased apoptosis, or inhibition of tumour growth^38, 39^. It could also lead to no detectable changes in cell viability, stemness or tumour growth as found in our studies and by others^27, 40^. Indeed, clinical trials with survivin inhibition have revealed that the anti-cancer efficacy of the inhibition of survivin is more pronounced when combined with chemotherapy^41–44^. This is in agreement with our finding that survivin silencing with the chimera was only effective against cancer stem cells when combined with 5-FU, resulting in reduced self-renewal and tumour growth and enhanced apoptosis (Figs 2–4). Therefore, our data suggest that survivin is one of the key players responsible for the innate chemoresistance of colorectal cancer stem cells. Taken together, our results indicate that targeting survivin in cancer stem cells in combination with chemotherapeutic drugs constitutes a new avenue to improve treatment outcome in oncologic clinics.

Chemotherapy is one of the important treatment strategies used for managing patients with cancer. For colorectal cancer, 5-FU and oxaliplatin are the major chemotherapeutic agents used by oncologists for treating colorectal cancer. Such drugs are known to be largely ineffective in killing cancer stem cells. Furthermore, innate chemoresistance is responsible for the drug-resistance frequently encountered in colorectal cancer patients. Our data presented here suggest that downregulation of survivin results in the reversal of the innate chemoresistance to both 5-FU and oxaliplatin in colorectal cancer stem cells (Figs 2, 3 and 4, Table 1). By combining targeted siRNA delivery with the treatment of 5-FU, we have transformed a standard chemotherapeutic drug known to be ineffective against cancer stem cells into an effective colorectal cancer stem cell killer. In addition to *in vitro* and *ex vivo* data which showed promising results for the combinatorial treatment of chimera and 5-FU in enhancing apoptosis and eliminating cancer stem cells, *in vivo* treatment of tumour-bearing mice using PEG-labeled chimera with 5-FU via tail vein injection resulted in enhanced tumour apoptosis and reduced tumour self-renewal (Figs 3 and 4). As shown in Fig. 4, the most prominent effect of the combinatorial treatment of aptamer-guided survivin siRNA and 5-FU occurred within the first 25 days of treatment. The limited number of chimera injections may have been insufficient in maintaining survivin downregulation in the HT-29 tumours throughout the duration of the combinatorial treatment. Additionally, the continuation of 5-FU treatments without the chimera potentially elicited pro-survival responses such as autophagy, contributing to drug resistance and tumour growth^45–47^. Thus, our results suggest that maintaining survivin downregulation simultaneously with chemotherapy may be required in order to enhance the anti-tumour efficacy and improved outcome in oncologic clinics.

In conclusion, EpCAM aptamer-guided delivery of survivin siRNA is able to target both the bulk and cancer stem cells in a xenograft colorectal cancer model. The aptamer-guided knockdown of survivin reverses the innate chemoresistance of colorectal cancer stem cells. The aptamer-mediated siRNA delivery to cancer stem cells *in vivo* opens a new avenue to target specific cancer cell populations and to modulate genes that are critical for cancer cell survival and chemoresistance.

## Material and Methods

### Animals

Female 6-week old immunodeficient NOD/SCID mice were purchased from the Animal Resources Centre (Perth, Australia). All experiments were performed in accordance with the Australian Code of Practice for the Care and Use of Animals for Scientific Purposes and Guidelines to Promote the Wellbeing of Animals Used for Scientific Purposes from Australian Government’s National Health and Medical Research Council. The Deakin University Animal Ethics Committee approved all experimental protocols.

### Cell culture

HT-29 (human colorectal adenocarcinoma, ATCC® HTB38™) cells were cultured in Dulbecco’s Modified Eagle Medium (DMEM) (Invitrogen, Australia) supplemented with 10% fetal bovine serum (FBS, Hyclone, Canada), 50 U/L penicillin and streptomycin (Invitrogen, Australia) and 1× Glutamax (Life Technologies). Cell cultures were kept in a humidified atmosphere with 5% CO_2_ at 37 °C. For aptamer-siRNA treatment, 20 nM of either chimeras were added to the cell culture for 48 and 24 hours for total protein and RNA extraction, respectively.

### Western analysis

Western analysis was performed as described in detail in our previous publication^27^. The primary antibodies used in the western blot were mouse anti-human β-actin (1:5000, Sigma) and mouse anti-human survivin (1:400, Cat. No: SC-17779, Santa Cruz). The secondary antibodies were HRP-conjugated goat anti-mouse secondary antibody (1:10000, Thermo Fisher) or goat anti-rabbit secondary antibody (1:5000, Pierce). Quantification of ECL signals was conducted using a LAS-4000 Imaging System (GE Healthcare Life Sciences) with β-actin as a loading control.

### RNA extraction and qrRT-PCR

Total RNA was extracted using Trizol (Invitrogen) following the manufacturer’s protocol, and cDNA was generated from the extracted RNA using High Capacity cDNA Reverse Transcription kit (Applied Biosystems). Fast SYBR Green Master Mix (Invitrogen) and Stratagene Mx3000 P system (Agilent Technologies) were used to carry out qRT-PCR assays with GAPDH as an internal control. The primers used for qRT-PCR assays were survivin (forward primer: 5′-GAACTGGCCCTTCTTGGAG-3′, reverse primer: 5′-AAGTCTGGCTCGTTCTCAGT-3′) and GAPDH (forward primer: 5′-GAAATCCCATCACCATCTTCCAGG-3′, reverse primer: 5′-GAGCCCCAGCCTTCTCCATG-3′).

### *In vitro* tumoursphere and *ex vivo* xenograft assays

The tumoursphere assay was performed in accordance with previously reported protocols^48^. Cells were collected by trypsinization and resuspended as single cells in cancer stem cell media containing serum-free DMEM/F12 (Invitrogen) supplemented with 100 units/mL B27 (Gibco), 10 µg/mL Insulin (Sigma), 20 ng/mL EGF (Sapphire Bioscience), and 20 ng/mL bFGF (Sapphire Bioscience). The cells were plated in a round-bottom 96-well ultra-low attachment plates (Corning) at densities of 1, 5, 20, and 50 cells/well. Sphere formation was recorded 5–7 days after incubation at 37 °C in a humidified atmosphere with 5% CO_2_. Cancer stem cell frequency was calculated using the limiting dilution software package (ELDA) on the website of Walter and Eliza Hall Institute of Medical Research (http://bioinf.wehi.edu.au/software/elda/index.html)^49^. Only spheres with a size larger than 50 µm in diameter were counted. For *ex vivo* xenograft assay, cells were injected subcutaneously into the left flanks of female NOD/SCID mice at three cell densities (5 × 10^4^, 1 × 10^4^, and 1 × 10^4^) in serum-free DMEM with Matrigel at 1:1 ratio. Tumours were detected by palpation.

### Mice treatment for *in vivo* tumoursphere and xenograft assays

Female NOD/SCID mice with HT-29 tumours (60 mm^3^) in the left flanks received 2 nmol of aptamer-siRNA chimera intravenously via the tail-vein on days 1, 3, and 5, and PBS on day 7, while mice with 5-FU treatment received PBS i.v. injection on day 1 and 30 mg/kg of 5-FU via on days 3, 5, and 7. The tumours were collected 48 hours after the final injection and dissociated into single cell suspensions to be used for *in vitro* tumoursphere assay (50, 20, 10, 5, 1 cells/well) and xenograft assay (1 × 10^5^, 1 × 10^4^, 1 × 10^3^, 1 × 10^2^ cells/mouse). The cancer stem cells frequency was measured using the limiting dilution software package on the website of Walter and Eliza Hall Institute of Medical Research (http://bioinf.wehi.edu.au/software/elda/index.html)^49^.

### Tumour dissociation

Tumours collected from xenografts were washed thoroughly with Hank’s buffer containing 1% penicillin/streptomycin to remove excess blood and any extraneous material. The tumours were placed in a sterile petri dish and minced into smaller pieces (approximately 2–4 mm^3^) with a scalpel. The chopped tissues were collected and rinsed with Hank’s buffer, then suspended in dissociation medium containing DMEM, 20% FBS, 2% penicillin/streptomycin, 100 units/mL B27, 10 µg/mL Insulin, 20 ng/mL EGF, 20 ng/mL bFGF, and 50 U/mL collagenase II (Sigma). The dissociation medium was used on the chopped tumours at a ratio of 6 ml per gram of tumour and incubated at 37 °C overnight on a rotating orbit mixer incubator. Cells were collected after incubation and centrifuged at 1,000 g for 5 minutes. The pellets were suspended and washed with PBS twice by centrifugation at 500 g to remove any residual debris.

### TUNEL assay

The dissociated tumour cells were washed with PBS and fixed on glass slides with 1% paraformaldehyde. Apoptotic cells were detected using TUNEL Apoptosis Detection Kit (Millipore) following the manufacture’s protocol. Fixed cells were counterstained with mounting medium (Vectashield, Vector Laboratories, Burlingame, CA) containing 1 µg/mL DAPI, and the cells were visualized with Fluoview FV10i laser scanning confocal microscope (Olympus, NSW, Australia).

### *In vivo* treatment for tumour growth rate and survival

Female NOD/SCID mice with HT-29 tumours (60 mm^3^) in the left flanks received 2 nmol of aptamer-siRNA chimera on days 1, 3, and 5, and PBS on days 7, 9, and 11, while mice with 5-FU treatment received intravenous injection of PBS on day 1 and 30 mg/kg of 5-FU via IV injections on days 3, 5, 7, 9 and 11. The weights and tumour dimensions of mice were monitored daily. The endpoints set for the experiments were defined by rapid weight loss by 20% of the body weight, tumour diameter exceeding 13 mm, and any health deteriorations impacting on the welfare of the animal as set by the Deakin University Animal Ethics Committee. Tumour diameters were measured with a digital caliper and tumour volume was calculated with the formula: Volume = Length × Width^2^/2.

## Electronic supplementary material

Supplementary Information
