# Author Correction: Aptamer-mediated survivin RNAi enables 5-fluorouracil to eliminate colorectal cancer stem cells

**DOI:** 10.1038/s41598-020-77490-4

**Published:** 2020-12-11

**Authors:** Hadi AlShamaileh, Tao Wang, Dongxi Xiang, Wang Yin, Phuong Ha-Lien Tran, Roberto A. Barrero, Pei-Zhuo Zhang, Yong Li, Lingxue Kong, Ke Liu, Shu-Feng Zhou, Yingchun Hou, Sarah Shigdar, Wei Duan

**Affiliations:** 1 School of Medicine and Centre for Molecular and Medical Research, Deakin University, 75 Pigdons Road, Waurn Ponds, VIC 3216 Australia; 2 School of Nursing, Zhengzhou University, Zhengzhou, 450001 Henan Province China; 3 Centre for Comparative Genomics, Murdoch University, 90 South Street, Murdoch, WA 6150 Australia; 4 Suzhou GenePharma, 199 Dongping Street, Suzhou, 215123 China; 5 Cancer Care Centre, St George Hospital and St George and Suthland Clinical School, University of New South Wales (UNSW), High Street, Kensington, NSW 2052 Australia; 6 Deakin University, Institute for Frontier Materials, 75 Pigdons Road, Waurn Ponds, VIC 3216 Australia; 7 College of Life Sciences, Sichuan University, No.24 South Section 1, Yihuan Road, Chengdu, 610041 China; 8 Department of Bioengineering and Biotechnology, College of Chemical Engineering, Huaqiao University, 668 Jimei Avenue, Xiamen, 361021 Fujian China; 9 Center for Qinba Region’s Sustainable Development, Shaanxi Normal University, No.199, South Chang’an Road, Xi’an, 710062 Shaanxi China

Correction to: *Scientific Reports* 10.1038/s41598-017-05859-z, published online 19 July 2017

This Article contains an error in the order of panels of Figure 1. Panels a, b, c, d and e should appear in the order d, e, a, b, c. The correct Figure appears below as Figure 1.

Additionally, the panels in Figures 3 and 4 are missing labels. The correct Figures appear below as Figures 2 and 3 respectively.

**Figure 1:**
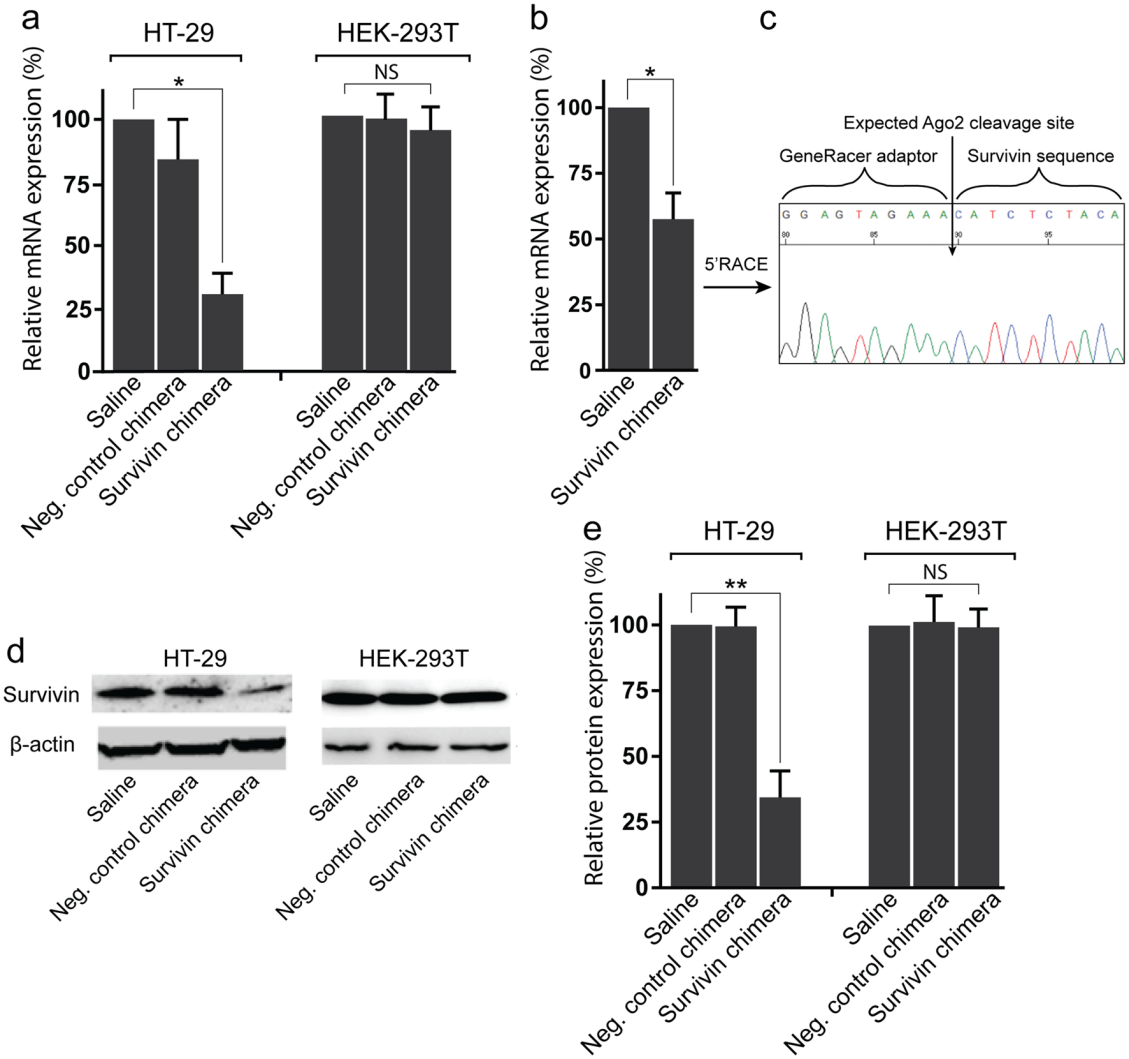
EpCAM aptamer-guided RNAi effectively silenced survivin. (**a**) Specificity and efficacy of EpCAMaptamer guided RNAi in knocking down survivin mRNA. Chimera or negative control chimera were incubated with HT-29 or HEK-2913T cells for 24 hours and the total RNA was extracted for qRT-PCR analysis of survivin mRNA levels. GAPDH was used as an internal control. (**b**,** c**) HT-29 Tumour-bearing mice were treated with 2 nmol/mouse of PEG-labelled chimera for 48 hours. The tumours were collected for RNA extraction followed by qRT-PCR analysis of survivin mRNA expression (**b**) and 5′RACE assay (**c**). (**d**) Effective downregulation of survivin protein via EpCAM aptamer-guided RNAi. Chimera or negative control chimera were incubated with HT-29 or HEK-2913T cells for 48 hours and the survivin protein levels were analyzed using Western blot analysis. β-actin was used as a loading control. (**e**) The bar graph shows the survivin protein levels in various treatment groups. Data shown are means ± SEM, n = 3. *p < 0.05, **p < 0.005. NS, no statistically significant difference..

**Figure 2:**
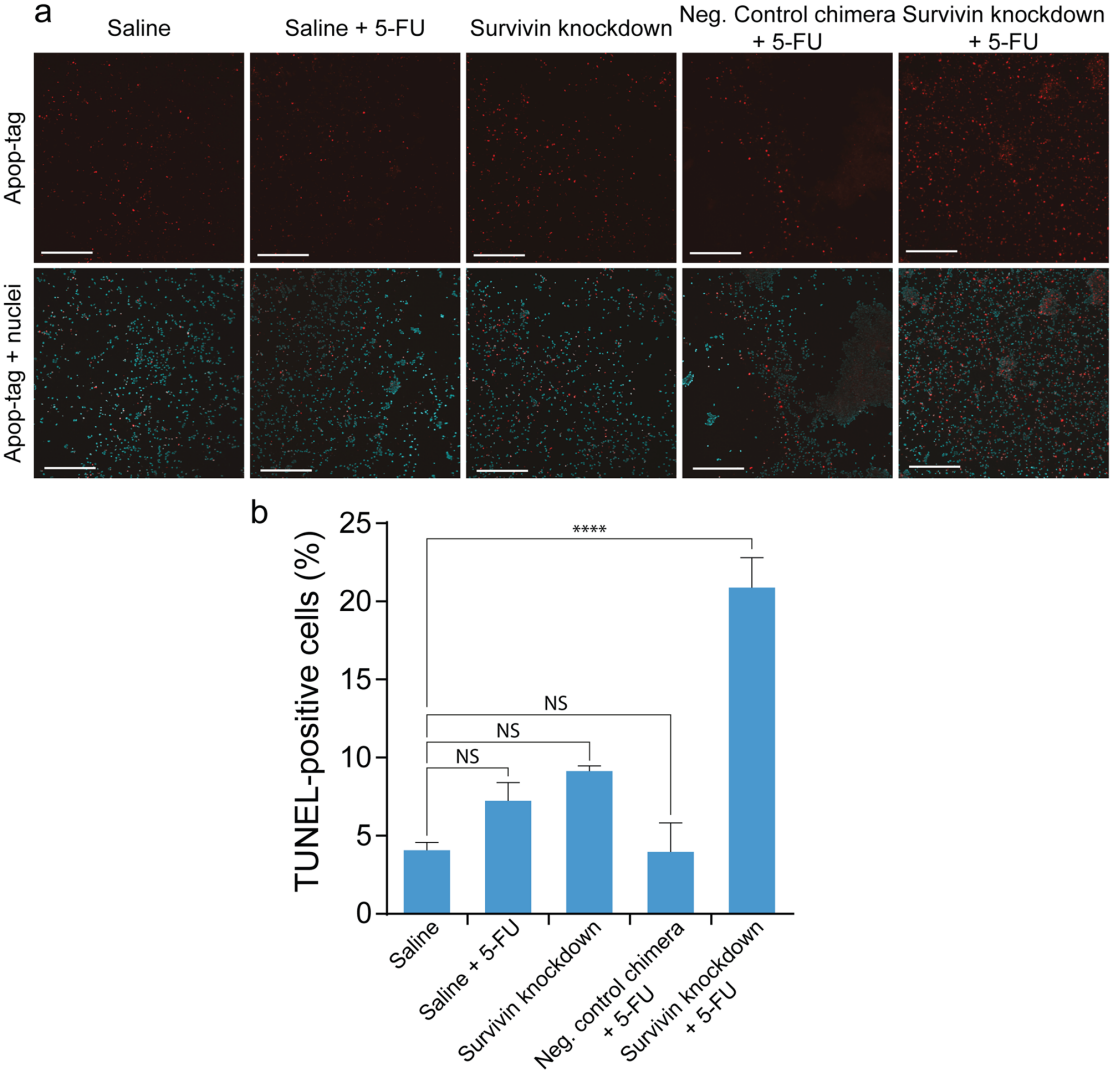
Survivin knockdown *in vivo* enhances 5-FU-induced apoptosis in HT-29 tumour cells. (**a**) Representative images of TUNEL apoptosis assay on dissociated HT-29 xenograft tumours after *in vivo* treatment with chimera and 5-FU. NOD/SCID mice bearing HT-29 tumours (60 mm^3^) were treated intravenously with 3 injections of 2 nmol/mouse of chimera with or without 3 additional treatment of 30 mg/kg of 5-FU. Two days after the final treatment, tumours were dissociated by collagenase digestion and subjected to TUNEL apoptosis assay. (**b**) Percentage of apoptotic cells in treated tumours. Data shown are means ± SEM, n = 3. ****p < 0.001. NS, no statistically significant difference.

**Figure 3:**
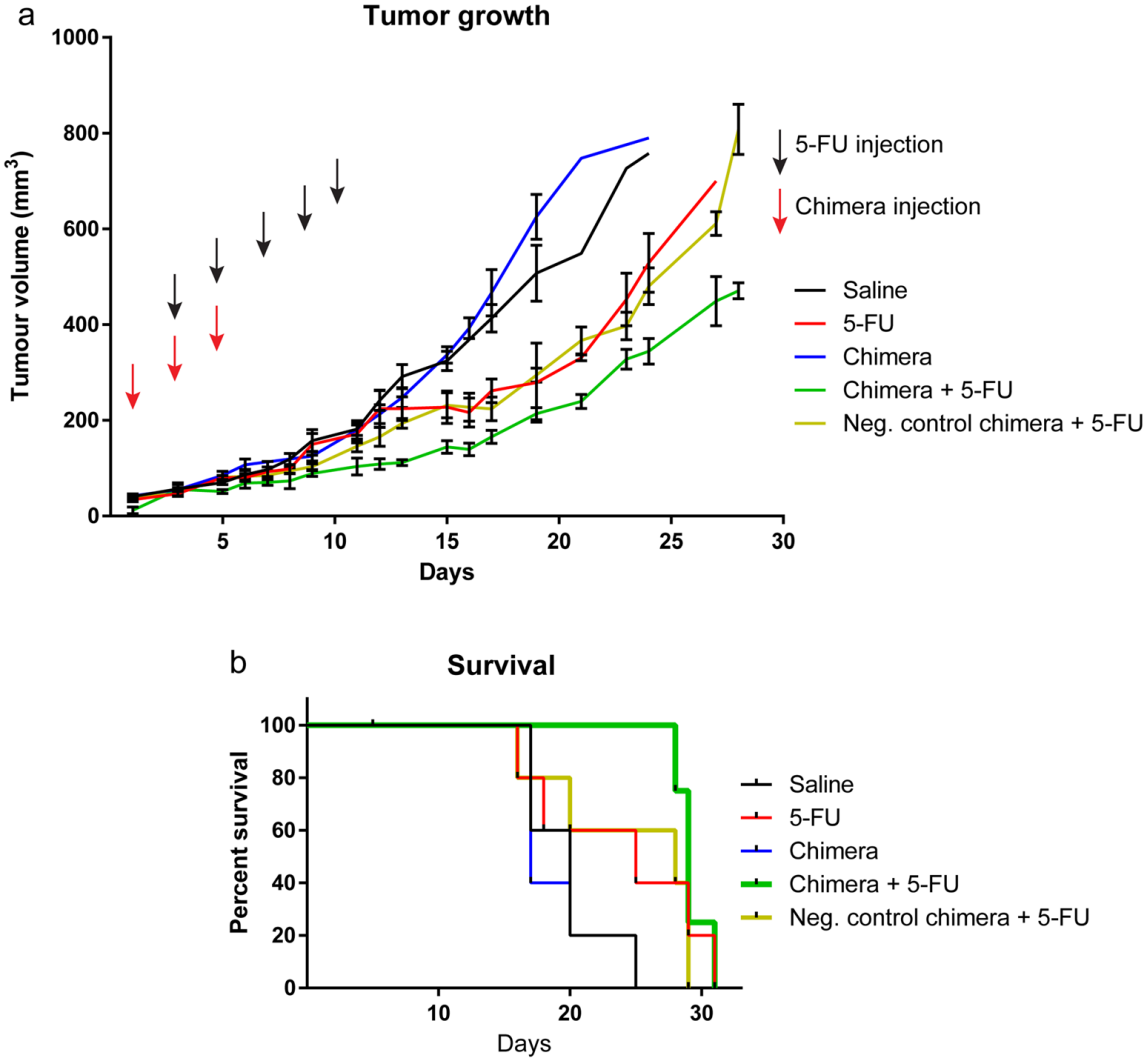
Combined chimera and 5-FU treatments improves therapeutic outcome in HT-29 tumour-bearing mice. (**a**) The graph represents the tumour volume in mice in response to various treatments as indicated. (**b**) Survival rate of mice observed over the course of various treatments. Data shown are mean ± SEM, n = 4–5.

Finally, the Supplementary Information file contains a typographical error.

“The chimera structure features an asymmetric structure to facilitate recognition by an endogenous Dicer enzyme to cleave the chimera which results in the release of the expected 21-mer siRNA sequence [251] (Figure S1b).”

should read:

“The chimera structure features an asymmetric structure to facilitate recognition by an endogenous Dicer enzyme to cleave the chimera which results in the release of the expected 21-mer siRNA sequence (Figure S1b).”

