# Supplementary material for: Aptamer-mediated survivin RNAi enables 5-fluorouracil to eliminate colorectal cancer stem cells
Source: Sci Rep. 2017 Jul 19;7:5898. doi: 10.1038/s41598-017-05859-z (PMC5517644; doi:10.1038/s41598-017-05859-z)
Supplement: Supplementary file 1 — Supplementary Information [file 41598_2017_5859_MOESM1_ESM.pdf]

Supplementary information

Aptamer-mediated survivin RNAi enables 5-fluorouracil to eliminate colorectal cancer stem cells

Hadi AlShamaileh[1*], Tao Wang[1,2*], Dongxi Xiang[1], Wang Yin[1], Phuong Ha-Lien Tran[1], Roberto A Barrero[3], Pei-Zhuo Zhang[4], Yong Li[5], Lingxue Kong[6], Ke Liu[7], Shu-Feng Zhou[8], Yingchun Hou[9], Sarah Shigdar[1], and Wei Duan[1**]

[1]School of Medicine and Centre for Molecular and Medical Research, Deakin University, 75 Pigdons Road, Waurn Ponds, Victoria 3216, Australia.

[2] School of Nursing, Zhengzhou University, Zhengzhou, Henan Province, China, 450001.

[3]Centre for Comparative Genomics, Murdoch University, 90 South Street, Murdoch, WA 6150, Australia.

[4]Suzhou GenePharma, 199 Dongping Street, Suzhou, China, 215123

[5]Cancer Care Centre, St George Hospital and St George and Suthland Clinical School, University of New South Wales (UNSW), High Street, Kensington, NSW 2052, Australia

[6] Deakin University, Australia, Institute for Frontier Materials, 75 Pigdons Road, Waurn Ponds, Victoria, 3216

[7]College of Life Sciences, Sichuan University, No.24 South Section 1, Yihuan Road, Chengdu, P. R. China, 610041

[8]Department of Bioengineering and Biotechnology, College of Chemical Engineering, Huaqiao University, 668 Jimei Avenue, Xiamen, Fujian 361021, China.

[9]Center for Qinba Region's Sustainable Development, Shaanxi Normal University, No.199, South Chang’an Road, Xi'an, Shaanxi 710062, China,

*These authors contributed equally to this work.

**Corresponding authors: School of Medicine, Deakin University, Waurn Ponds, Victoria 3216, Australia. Tel.: +61 - 3 5227 1149; Fax: +61 - 3 5227 2945; wduan@deakin.edu.au

The supplementary information includes:

Supplementary Figure - 1. Aptamer-siRNA chimera structure.

Supplementary Figure - 2. Representative images for *in vitro* tumoursphere assay.

Supplementary Figure - 3. Targeting survivin enhances oxaliplatin efficacy against HT-29 colorectal cancer stem cells.

Supplementary Figure - 4. Tumour size and weights.

Supplementary Figure - 5. Combinatorial treatment with chimera and 5-FU of mice bearing xenograft tumours led to the inhibition of self-renewal of HT-29 cells.

Supplementary Figure - 6. Increased apoptosis of xenograft HT-29 cancer cells elicited by the combinatorial treatment of chimera and 5-FU.

Supplementary Figure - 7. Inhibition of xenograft tumour cell proliferation after the combinatorial treatment using chimera and 5-FU.

Table S1. Efficacy of *in vitro* survivin knockdown with or without 5-FU treatment in HT-29 tumour-formation as determined in a limiting dilution assay.

Supplementary Figure 1. Aptamer-siRNA chimera structure

The aptamer-siRNA chimera was designed by linking the EpCAM aptamer with a survivin-specific siRNA sequence (Figure S1a). For *in vivo* applications, a 20-kDa PEG was linked to the chimera to increase circulatory half-life. The chimera structure features an asymmetric structure to facilitate recognition by an endogenous Dicer enzyme to cleave the chimera which results in the release of the expected 21-mer siRNA sequence [251] (Figure S1b). As control, we used an alternative from of the chimera similar to the original but with 2 point mutations introduced in the siRNA sequence as shown in Figure S1a. These modifications abolished the survivin silencing efficacy but retained the aptamer-binding property, therefore controlling potential immune response-associated gene suppression and EpCAM-binding side-effects.

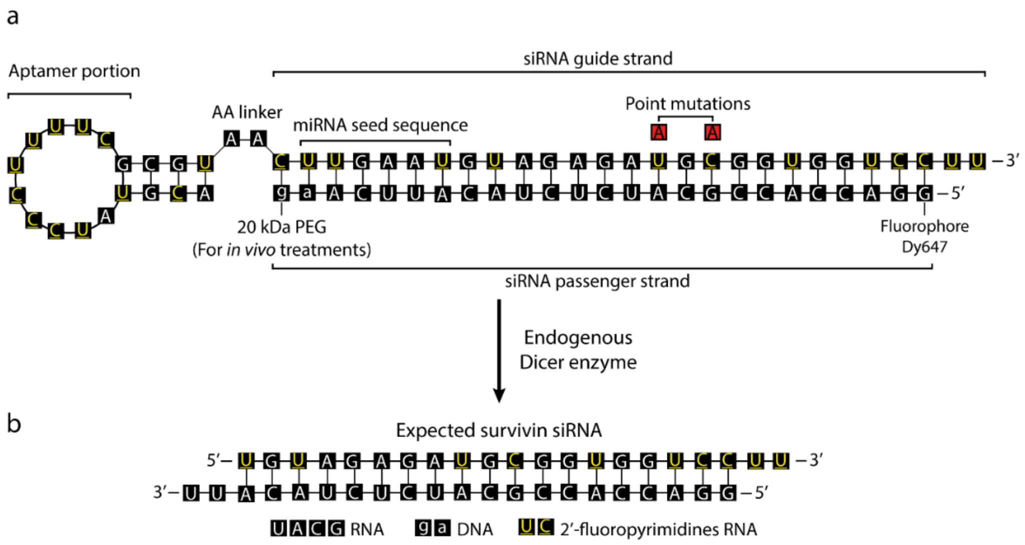

Figure S1. Schematic illustration of the aptamer-siRNA chimera. (a). Complete structure of the chimera comprised of an aptamer (binding portion) and an RNA duplex (siRNA portion). (b). Upon cell binding and receptor-mediated endocytosis, the chimera is recognized by the endogenous Dicer enzyme and subsequently integrated into the RNAi machinery, leading to the release of the predicted 21-mer survivin siRNA.

Supplementary Figure - 2. Representative images for *in vitro* tumoursphere assay.

To measure tumour self-renewal, HT-29 cells were treated with 20 nM of the chimera or negative control chimera for 48 hours prior to plating on a 96-well or 6-well ultra-low attachment round bottom plates with or without 2 µM 5-FU at densities of 20, 10, and 5 cells per well. Figure S3 shows representative light microscopy images of tumourspheres in response to various treatments after 5 days of incubation at 37 °C.

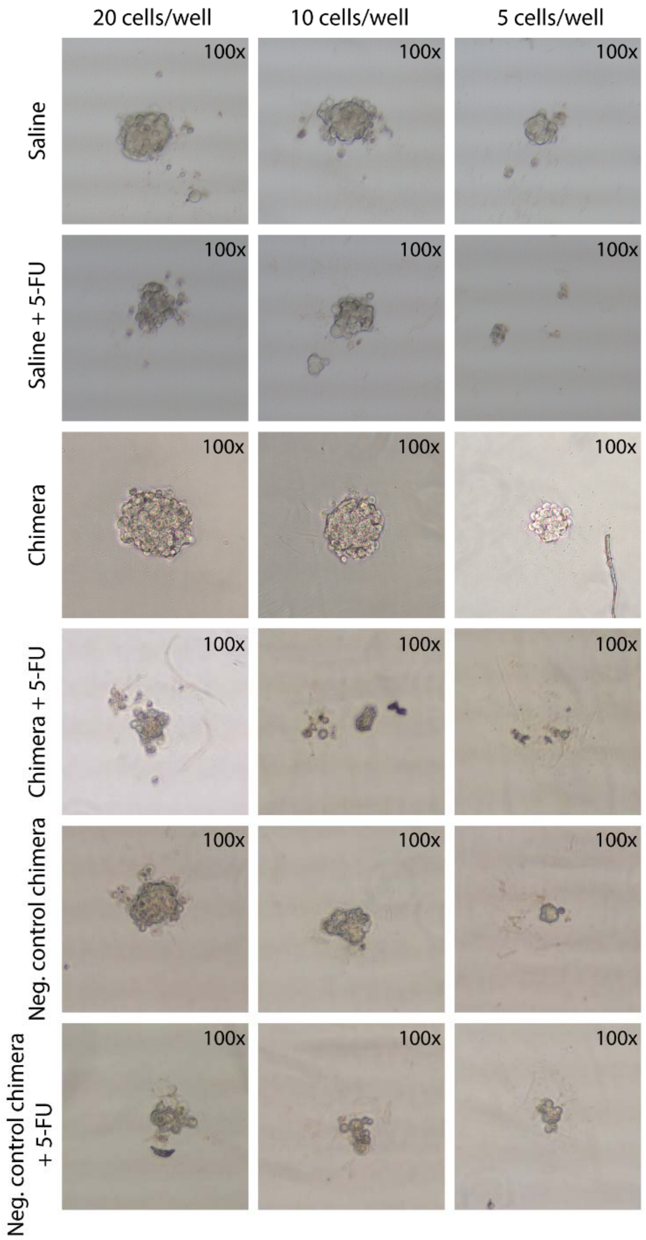

Figure S2. Representative images of in vitro tumoursphere assay. A tumoursphere-forming assay in 96-well plates was used to evaluate the potential of a cell to behave as a stem cell, in which a cell sphere with a well-defined border and a diameter larger than 50 µm was considered to be a tumourpshere. Images shown are at 100 x magnification.

Supplementary Figure 3. Targeting survivin enhances oxaliplatin efficacy against HT-29 colorectal cancer stem cells

HT-29 cells were treated with the chimera for 48 hours before trypsinization to prepare single cell suspension. The cells were plated into a round-bottom 96-well ultra-low attachment plates (Corning) with or without 50 nM oxaliplatin at densities of 1, 5, and 20 cells/well. As shown in Figure S2, there were no significant changes in self-renewal compared to saline control when the cells were treated with either survivin silencing or oxaliplatin. However, oxaliplatin treatment after survivin silencing resulted in approximately 2.7-fold decrease in self-renewal compared to saline control or oxaliplatin mono-treatment.

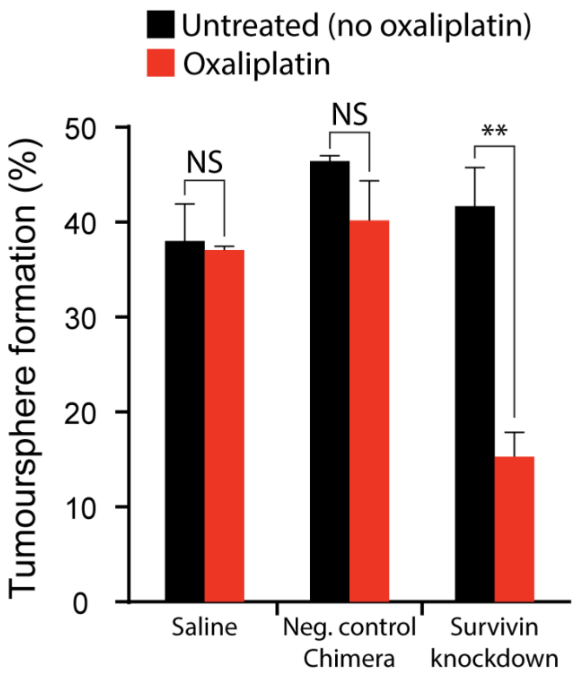

Figure S3. Survivin knockdown enhances oxaliplatin efficacy against HT-29 colorectal cancer stem cells. Survivin expression in HT-29 cells were knocked-down and plated in cancer stem cell media with or without oxaliplatin (50 nM) to evaluate their self-renewal capacities. The bar graph shows the estimated stem cell frequencies in each treatment groups. Data shown are means ± SEM, n = 3. ** $p < 0.01$.

Supplementary Figure - 4. Tumour size and weights.

Tumour-bearing NOD/SCID mice underwent intravenous treatments with 2 nmol/mouse of chimera or negative control chimera on days 1, 3, and 5 with or without 30 mg/kg of 5-FU on days 3, 5, and 7. Forty-eight hours after final treatment, tumours were extracted for size comparison (Figure S4a) and weighed individually (Figure S4b).

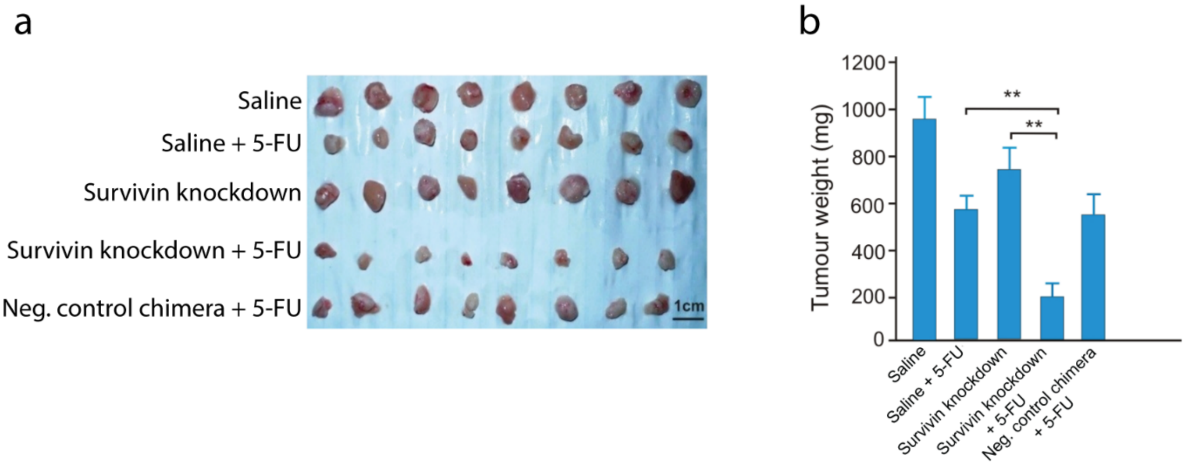

Figure S4. Combinatorial survivin knockdown with 5-FU treatment reduces tumour weight. (a) Tumours from NOD/SCID mice were laid out for comparison. Scale bar = 1 cm. **(b)** Extracted tumours were weighed individually. Data shown are mean ± SEM, n = 8. ** $p < 0.01$.

Supplementary Figure - 5. Combinatorial treatment with chimera and 5-FU of mice bearing xenograft tumours led to the inhibition of self-renewal of HT-29 cells.

After i*n vivo* treatment of tumour-bearing mice, the extracted tumours were dissociated into single-cell suspension with collagenase, and the dissociated cells were plated into 96-well low-attachment round bottom plates at various cell densities. After 5 days of incubation at 37 °C, each tumoursphere was examined with light microscopy. Figure S5 shows representative images of each tumoursphere.

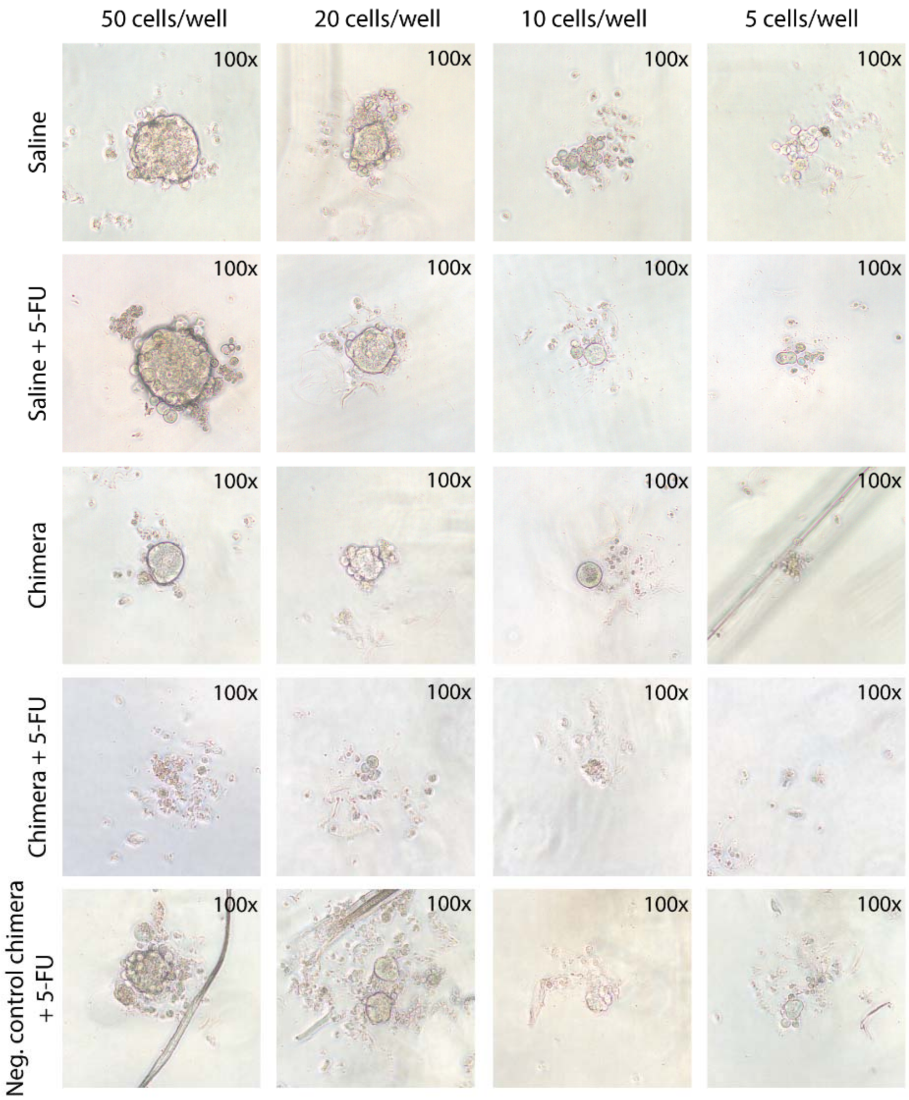

Figure S5. Representative images of tumourspheres of xenograft tumour cells after *in vivo* treatment. Dissociated xenograft tumours were used in tumoursphere-forming assay to detect cells that behave as stem cells. Only cells with well-defined borders and a diameter larger than 50 µm were considered as tumourspheres. Images shown are at 100 x magnification.

Supplementary Figure - 6. Increased apoptosis of xenograft HT-29 cancer cells elicited by the combinatorial treatment of chimera and 5-FU.

To measure the efficacy of survivin knockdown with the chimera in combination with 5-FU treatment in the induction of tumour cell apoptosis *in vivo*, tumours from *in vivo*-treated NOD/SCIFD mice were paraffin embedded and formalin fixed. The tumour sections were then prepared for TUNEL assay. As shown in Figure S4, combinatorial treatment of survivin knockdown using the chimera and 5-FU resulted in 200% more apoptosis compared with 5-FU treatment with or without the negative control chimera.

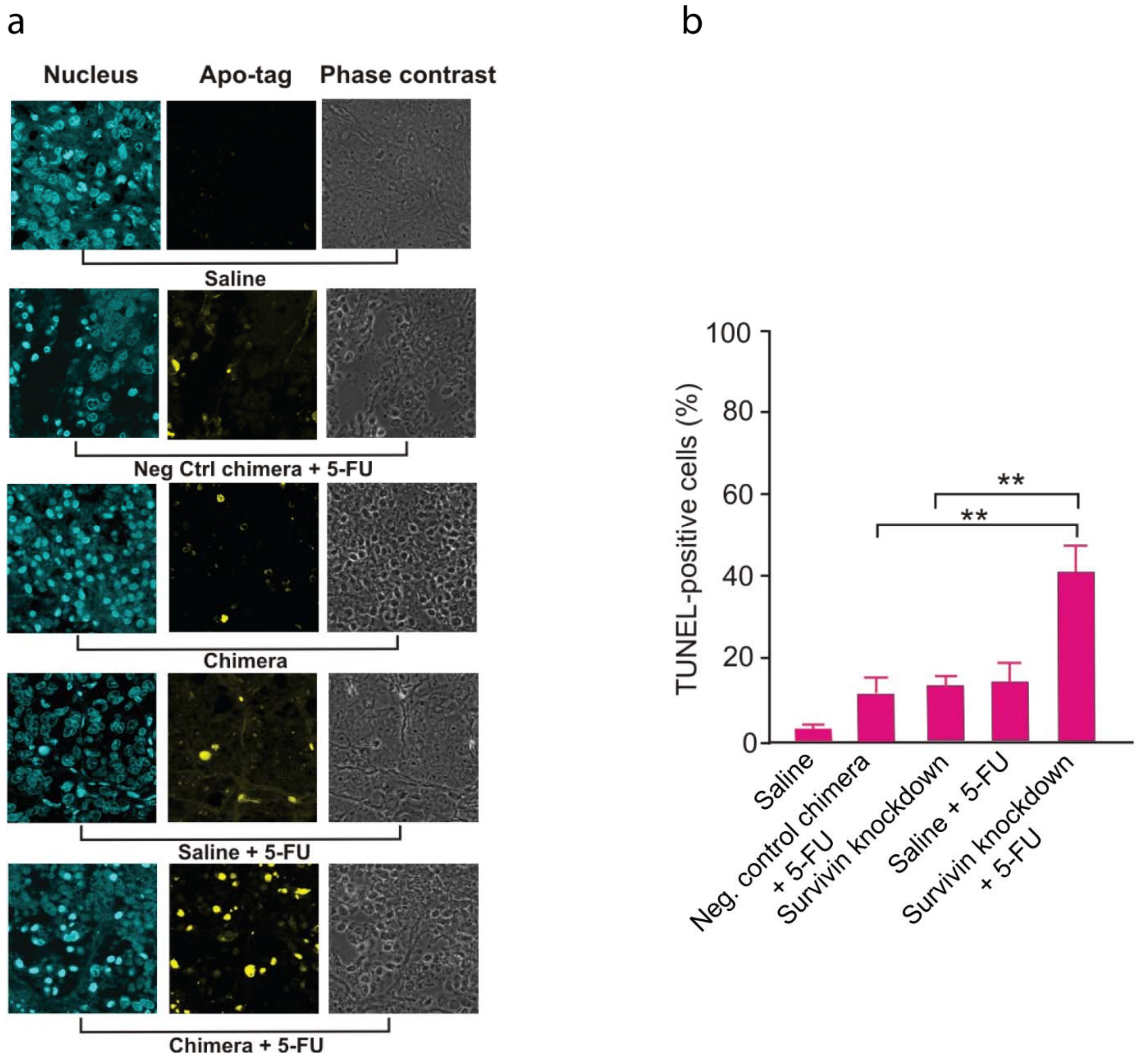

Figure S6. *In vivo* treatment of tumour-bearing NOD/SCID mice with survivin knockdown and 5-FU enhances tumour cell apoptosis. (a) Representative images of TUNEL apoptosis assay on tumour sections after *in vivo* treatment with chimera and 5-FU. **(b)** Percentage of TUNEL-positive cells in treated tumours. Data shown are means ± SEM, n = 8. ** $p < 0.01$.

Supplementary Figure - 7. Inhibition of xenograft tumour cell proliferation after the combinatorial treatment using chimera and 5-FU.

HT29 xenograft tumours were extracted 48 hours after the completion of intravenous treatment of tumour-bearing NOD/SCID mice with 2 nmol/mouse of the chimera or negative control chimera with or without 30 mg/kg of 5-FU. Tumour sections were prepared to study tumour cell proliferation with the Ki-67 marker. Compared to saline control, treatment with 5-FU, chimera, or negative control chimera with 5-FU led to approximately 2-fold reduction in Ki-67-positive cells. Furthermore, the combinatorial treatment of chimera with 5-FU led to a 4-fold reduction in Ki-67-positive cells, indicating the effective inhibition of tumour cell proliferation.

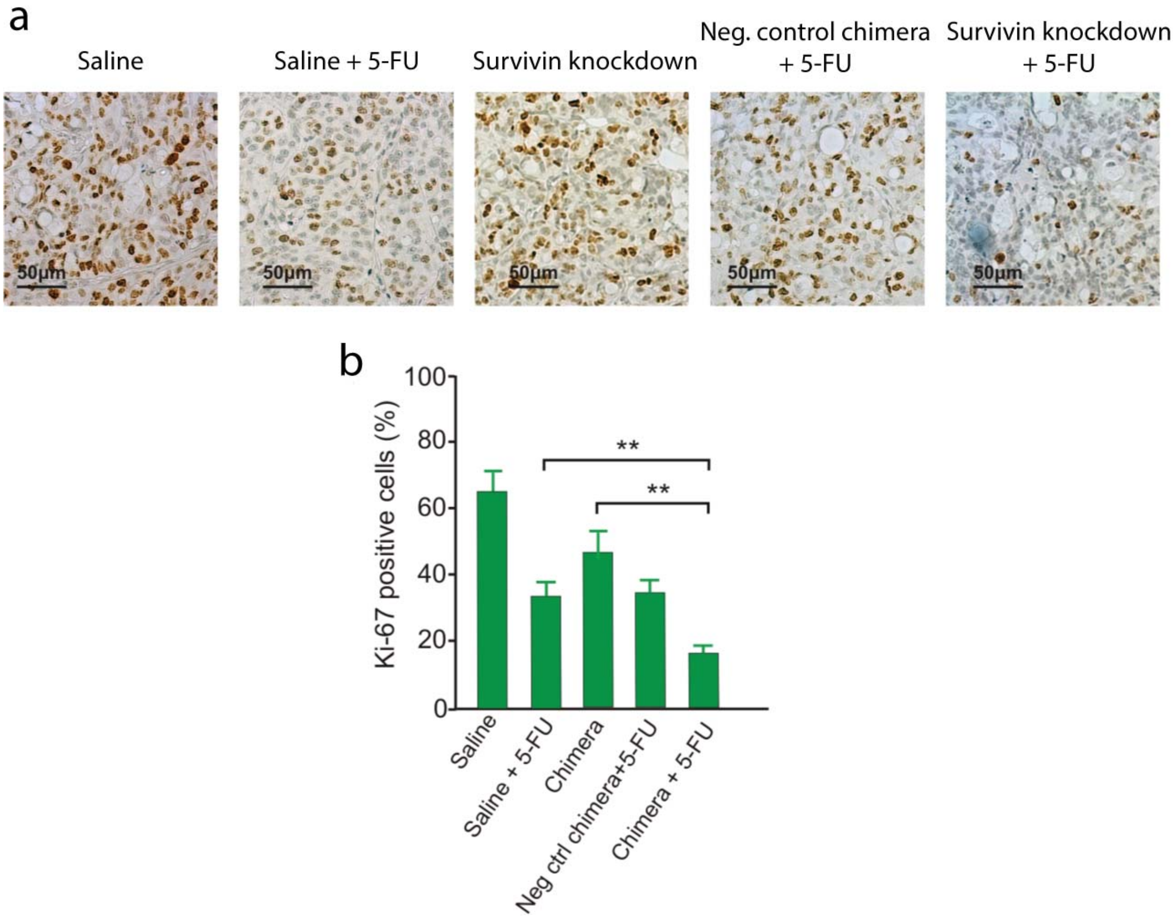

Figure S7. ***In vivo*** **treatment of tumour-bearing NOD/SCID mice with survivin knockdown and 5-FU inhibits tumour cell proliferation. (a)** Representative images of Ki-67 staining after *in vivo* treatment of tumour-bearing mice. **(b)** Percentage of KI-67-positive cells in treated tumours. Data shown are means ± SEM, n = 8. ** $p < 0.01$.

Table S1| Efficacy of *in vitro* survivin knockdown with or without 5-FU treatment in HT-29 tumour-formation as determined in a limiting dilution assay.

Treatment group	Cell dose per injection	Tumour incidence	Latency (days)	Estimated stem cell frequency	95% confidence interval
Saline	5×10^4	4/4	12 – 18	100%	0.047-100%
	1×10^4	4/4	18 – 22		
	1×10^3	4/4	18 – 35		
Saline + 5-FU	5×10^4	4/4	18 – 35	100%	0.047-100%
	1×10^4	4/4	15 – 49		
	1×10^3	4/4	35 – 49		
Neg. chimera control + 5-FU	5×10^4	4/4	18 – 22	100%	0.047-100%
	1×10^4	4/4	25 – 35		
	1×10^3	4/4	35 – 92		
Chimera	5×10^4	4/4	12 – 18	100%	0.047-100%
	1×10^4	4/4	18 – 22		
	1×10^3	4/4	18 – 35		
Chimera + 5-FU	5×10^4	4/4	35 – 56	0.005%	0.002-0.013
	1×10^4	4/4	49 – 61		
	1×10^3	0/4	–		
